# Therapeutic Applications of Natural Products in Biomedicine and Pharmacotherapy

**DOI:** 10.3390/life16060873

**Published:** 2026-05-22

**Authors:** Ashok Kumar Sah, Sakshi Patel, Rahul Kumar, Prem Shankar Mishra, Rakhi Mishra, Abdulkhakov Ikhtiyor Umarovich, Rabab H. Elshaikh, Shagun Agarwal, Ashwani Bhardwaj, Ranjay Kumar Choudhary, Ayman Hussein Alfeel

**Affiliations:** 1 Department of Medical Laboratory Science, College of Applied and Health Sciences, A’ Sharqiyah University, Ibra 400, Oman; ashok.sah8@gmail.com (A.K.S.); rabab.mahmoud@asu.edu.om (R.H.E.); ranjay.choudhary@asu.edu.om (R.K.C.); 2 Faculty of Pharmacy, Vidya University, Meerut 250005, UP, India; sakshi.p.090901@gmail.com (S.P.); rk.aumclass223@gmail.com (R.K.); 3 Noida Institute of Engineering and Technology (Pharmacy Institute), Greater Noida 201306, UP, India; rakhi.misra84@rediffmail.com; 4 Department of Faculty and Hospital Therapy, Bukhara State Medical Institute, Bukhara 200100, Uzbekistan; mironsho@rambler.ru; 5 School of Allied Health Sciences, Galgotias University, Greater Noida 203201, UP, India; shagunmpt@gmail.com; 6 Department of Medical Laboratory Technology, Kalpana Chawala Govt Polytechnic for Women, Ambala City 134003, HR, India; bhardwaj.ashwani58@gmail.com; 7 Department of Medical Laboratory Sciences, College of Health Sciences, Gulf Medical University, Ajman 4184, United Arab Emirates

**Keywords:** natural products, pharmacotherapy, semisynthetic derivatives, alkaloids, flavonoids, terpenoids, polyketides, glycosides, drug discovery, genome mining, synthetic biology, precision medicine, ADMET

## Abstract

Natural products are the fundamentals of drug discovery due to their exceptional structural diversity and biological activity’s evolutionary optimization. The review provides a critical and integrative analysis of natural products in pharmaceutical chemistry, highlighting their significance for current biomedicine and pharmacotherapy. The review is organized around a system that connects structure, function, and translation, focusing on structural analysis, scaffold design, and mechanistic understanding in major disease-relevant therapeutic areas. Investigations on representative compounds like paclitaxel, artemisinin, and curcumin are presented to explain the way molecular architecture regulates pharmacological activity, drug selectivity, and clinical performance. The review evaluates significant medicinal chemistry strategies, including semisynthetic modification, prodrug design, and scaffold optimization, and their crucial roles in enhancing potency, pharmacokinetics, and safety. We critically examine the latest advancements in drug delivery technologies, particularly those based on nanotechnology and carrier-free methods, regarding their translational potential and regulatory concern. Current challenges pertaining to pharmacokinetics and ADMET properties, as well as the standardization of analysis, are also examined, emphasizing their impact on reproducibility in research. Researchers investigate the role and limitations of emerging fields such as genome mining, synthetic biology, and network pharmacology in enhancing discovery pipelines. Thus, this review integrates chemical, pharmacological, and translational approaches and suggests an effective strategy to overcome challenges in the development of natural products as the next generation of precision medicine therapeutic agents.

## 1. Introduction

Historically, natural products (alkaloids, terpenoids, polyphenols, etc.) have been an inexhaustible source for the discovery of therapeutic agents, and exhibit unparalleled chemical diversity, stereochemical complexity, and biological relevance [1]. Although the field of combinatorial chemistry and high-throughput screens continues to develop at an incredibly fast rate, natural products and their analogs still represent a large portion of drugs that are brought to market, especially in oncology, infectious diseases, and metabolic diseases [2]. The long-term pharmacological importance of natural products is illustrated by structurally complex scaffolds like paclitaxel, vincristine, artemisinin, and morphine.

**Figure 1:**
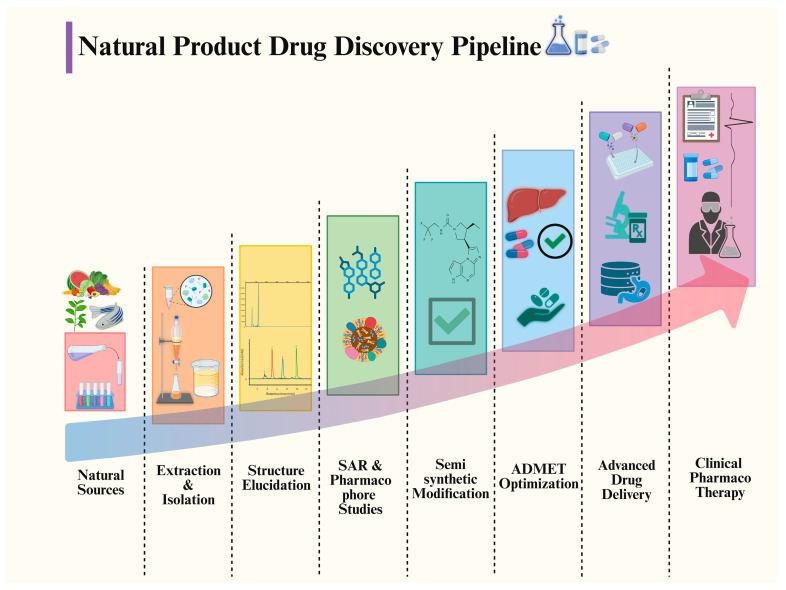
Pathway for the development of natural products. Abbreviations: SAR (Structure–Activity Relationship); ADMET (Absorption, Distribution, Metabolism, Excretion, and Toxicity).

According to pharmaceutical chemistry, natural products are chemically unique, having high levels of sp^3^ carbon, numerous chiral centers, rigid ring systems, and a wide variety of functional groups, all of which are associated with amplified target selectivity and binding affinity [3]. These characteristics often result in privileged scaffolds with the ability to modulate biologically relevant macromolecules encompassing enzymes, receptors, ion channels, and nucleic acids [4]. Moreover, natural products often become a lead compound or a natural compound that can be chemically optimized by semisynthetic modification to increase the potency and pharmacokinetic properties while reducing the drug toxicity profile [5]. In modern drug discovery efforts, natural products are not merely utilized as direct therapeutic agents but also as guides to synthetic analogs and pharmacophores as well as fragment-based drug development [6]. From sourcing to clinical assessment, the natural product-based drug development pipeline consists of many steps, as shown in Figure 1. Interest in natural products as multi-target agents, and thus able to treat complex diseases like cancer, neurodegeneration, and metabolic syndromes, was renewed by the combination of medicinal chemistry with computational modeling and systems pharmacology. Therefore, it is timely and necessary to conduct a systematized and chemistry-driven analysis of natural products in biomedicine and pharmacotherapy [7].

The practice of medicine with natural products can be traced back to thousands of years, and it is the foundation of the traditional systems of medicine like Ayurveda, Traditional Chinese Medicine, and Greco-Arabic medicine [8]. Initial therapeutic agents were sourced directly, in crude or semi-purified form, directly out of plants, microbes, and the sea [9]. The 19th-century isolation of pure bioactive compounds became a turning point in pharmaceutical chemistry, with examples being the discovery of quinine in Cinchona bark and of salicylic acid, which was subsequently used as the basis of aspirin [10]. The golden age of natural product drug discovery was in the mid-20th century when antibiotics like penicillin and streptomycin were discovered and changed the way infectious diseases were treated [11]. At the same time, the development of fermentation technology, chromatographic separation, and structural elucidation facilitated the interaction of structurally advanced secondary metabolites such as macrolides, polyketides, and non-ribosomal peptides.

The latter appearance of the methods of synthetic chemistry and combinatorial strategies in the late 20th century caused a temporary diversion of attention from natural products. However, limitations such as poor structural diversity and less-than-ideal pharmacokinetic properties of entirely synthetic libraries led to the resurrection of interest in natural products being biologically validated chemical entities [12]. Genome mining, metabolic engineering, and synthetic biology are now incorporated in modern approaches to open up an ever-expanding chemical space in which drugs can be discovered by accessing natural product scaffolds previously inaccessible [13].

The article is intended to be a critical narrative review with a systematic medicinal chemistry approach, which combines chemical, pharmacological and translational aspects of natural products. The extensive literature review was based on significant scientific databases, such as PubMed, Scopus, and Web of Science, and concentrated on the publications of the past two decades (2005–2025) but also included the classic earlier works of outstanding historical and scientific interest.

The search strategy included the use of keywords, which were natural products, pharmacotherapy, structure–activity relationship, drug delivery, ADMET, and synthetic biology. The studies were chosen according to their relevance to therapeutic use, mechanistic understanding, chemical structure–function interactions, and translatability. To ensure analytical rigor, only mechanistic or pharmacological articles were included.

This review, in contrast to the existing reviews, which focus on separate elements, e.g., classification, pharmacology, delivery systems, etc., has a structure–function-translation format, to connect molecular architecture and pharmacological activity, drug development strategies, and clinical applicability. This methodology offers an integrated viewpoint between medicinal chemistry design, biological processes, and translational issues thus providing some value to the traditional descriptive reviews in the discipline.

Natural products and their derivatives continue to be one of the key players in modern pharmacotherapy in a broad range of fields of therapy [14]. Microtubule stabilizers and topoisomerase inhibitor-derived products of natural origin, such as docetaxel and etoposide, remain integral to chemotherapeutic protocols in oncology [15]. Likewise, natural product scaffolds, such as β-lactams, tetracyclines and glycopeptides, are important to antimicrobial therapy and most of them have been optimized semi-synthetically. In addition to their direct pharmacological uses, natural products are considered as molecular probes for elucidating the biology pathway and as a molecular template for rational drug design [16]. Polyphenolic compounds like resveratrol and curcumin exhibit pleiotropic biological activities such as antioxidant, anti-inflammatory and epigenetic modulation, putting forward their potential as multi-target therapeutic agents [17].

Moreover, technological breakthroughs in the areas of pharmacokinetics, drug delivery through nanotechnology and prodrug approaches have boosted the clinical potential of naturally occurring products that previously showed low bioavailability or stability [18]. The combination of the structure–activity relationship (SAR) analysis, computer modeling, and high-resolution structural biology has allowed the optimization of natural product scaffolds in a precise manner resulting in increased efficacy and decreased toxicity [19]. Since the problem of multifactorial and chronic diseases is on the rise, the multilateral interaction of natural products with various biological targets makes them good candidates in network pharmacology and personalized medicine, particularly for conditions such as cancer, diabetes, and cardiovascular diseases [20]. Thus, pharmaceutical chemistry-based reviews of natural products in the fields of biomedicine and pharmacotherapy need to be done, in depth, to inform further drug discovery and development in the future.

## 2. Classification of Natural Products: Chemical and Biosynthetic Perspectives

**Figure 2:**
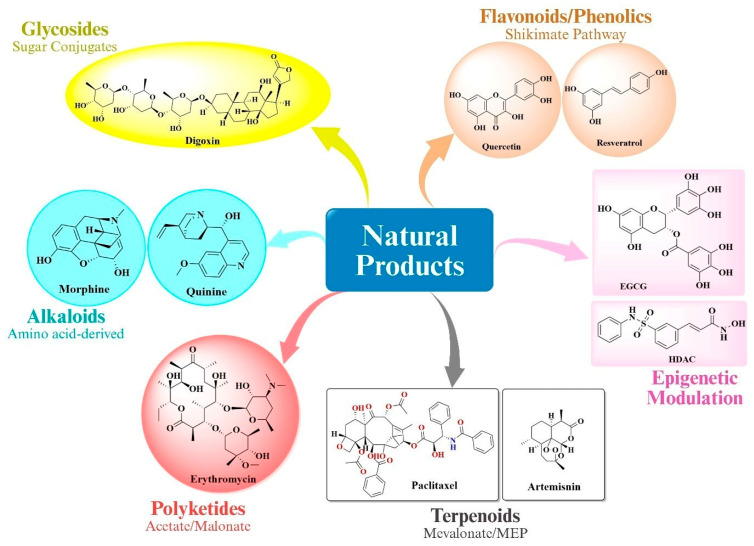
Chemical classification and biosynthetic origin of natural products. Abbreviations: MEP: Methylerythritol phosphate; HDAC: Histone Deacetylase; EGCG: Epigallocatechin gallate.

Natural products may be categorized using various conventional methods, such as pharmacological activity, structural class, or biosynthetic origin [21]. However, such discrete categorization schemes often overlook the functional link between molecular structure, biological activity, and clinical translation. Thus, an integrated medicinal chemistry framework that incorporates biosynthetic origin, core chemical scaffold, preferred pharmacophore traits, therapeutic relevance, and translational restrictions is used in this study to classify natural compounds [22]. Table 1 describes the key natural products categories with emphasis on the representative bioactive chemicals, structural frameworks and therapeutic potential [23]. This multidimensional approach enables a more methodical understanding of how structural characteristics influence biological interactions and pharmacological consequences. Additionally, it offers a logical foundation for integrating drug research and development procedures with the chemistry of natural products as shown in Figure 2. As a result, each class that is covered here is examined in terms of its structure and origin as well as its functional and translational relevance [24,25].

### 2.1. Alkaloids

- Origin of biosynthesis: Amino acids like tryptophan, tyrosine, ornithine, and lysine are the main sources of alkaloids [26].
- Chemical scaffold core: Nitrogen-containing heterocyclic frameworks, such as indole, isoquinoline, tropane, and purine systems, are what define them [27].
- Advantageous pharmacophore characteristics: Strong ionic contacts and π–π stacking with biological targets, including receptors and enzymes, are made possible by the presence of protonatable nitrogen atoms, aromatic π systems, and specified stereochemistry [28].
- Relevance to therapy: Alkaloids have various pharmacological properties, such as analgesic (morphine), antimalarial (quinine), and anticancer (vinblastine) effects [29].
- Principal restrictions: Toxicity, a restricted therapeutic index, and difficulties with synthesis or extraction often restrict their clinical application.

### 2.2. Isoprenoids

- Origin of biosynthesis: The mevalonate (MVA) or methylerythritol phosphate (MEP) routes are used to biosynthesize terpenoids from isoprene (C5) units [30].
- Chemical scaffold core: They are made up of repeated isoprene units that form linear or polycyclic hydrocarbon frameworks [31].
- Advantageous pharmacophore characteristics: Their action is mostly dependent on hydrophobic polycyclic cores, epoxide groups, and oxygenated functions like alcohols and peroxides.
- Relevance to therapy: Terpenoids with strong biological action, such as paclitaxel (anticancer) and artemisinin (antimalarial), are clinically significant [32].
- Principal restrictions: They often have restricted bioavailability, poor water solubility, and formulation difficulties.

### 2.3. Phenolics and Polyphenols

Polyphenols include several subclasses such as flavonoids, stilbenes, lignans, and phenolic acids.

- Origin of biosynthesis: The phenylpropanoid and shikimate pathways are the primary sources of these chemicals [33].
- Chemical scaffold core: They often have conjugated systems of aromatic rings with one or more hydroxyl groups [34].
- Advantageous pharmacophore characteristics: Metal chelation, radical scavenging, and hydrogen bonding are all made easier by phenolic hydroxyl groups.
- Relevance to therapy: Resveratrol and other phenolic compounds have anti-inflammatory, antioxidant, and epigenetic modulatory properties [35].
- Principal restrictions: Low bioavailability, quick metabolism, and restricted membrane permeability limit their use.

Flavonoids represent one of the largest and most pharmacologically important subclasses of polyphenols.

### 2.4. Flavonoids

Flavonoids are biosynthesized through the combined action of the shikimate pathway and the malonate (polyketide) pathway via phenylpropanoid intermediates.

- Origin of biosynthesis: Derived from the production of phenylpropanoids [36].
- Chemical scaffold core: Their structure is C6–C3–C6, with a heterocyclic pyran ring connecting two aromatic rings.
- Planar aromatic systems and numerous hydroxyl groups enable interaction with enzymes, receptors, and nucleic acids, providing advantageous pharmacophore characteristics [37].
- Relevance to therapy: Quercetin and other flavonoids have anti-inflammatory, antioxidant, and enzyme-inhibiting qualities [38].
- Principal restrictions: They are often constrained by low systemic availability, quick metabolic breakdown, and inadequate absorption [39].

### 2.5. Glycosides

- Origin of biosynthesis: Made by combining sugar parts with different aglycone structures, such as terpenoids, phenolics, and steroids.
- Chemical scaffold core: Made up of an aglycone (non-sugar moiety) connected to a glycone (sugar) [40].
- The glycosidic bond influences solubility, stability, and receptor engagement, which are all advantageous pharmacophore characteristics [41].
- Relevance to therapy: Digoxin, a cardiotonic, and salicin, an anti-inflammatory, are two examples.
- Principal restrictions: They often have varied pharmacokinetics, a limited therapeutic index, and hydrolytic instability.

### 2.6. Polyketides and Macrolides

- Origin of biosynthesis: Produced by polyketide synthases by the condensation of acetate and propionate molecules.
- Chemical scaffold core: Characterized by aromatic polyketide structures and macrocyclic lactones [42].
- Advantageous pharmacophore characteristics: Activity depends on lactone rings, ketone groups, and glycosidic substitutions.
- Relevance to therapy: Add essential medications like doxorubicin (anticancer) and erythromycin (antibiotic) [43].
- Principal restrictions: Toxicity, the development of resistance, and metabolic instability are among the limitations.

### 2.7. Peptides and Depsipeptides

- Origin of biosynthesis: Derived from both non-ribosomal and ribosomal routes of peptide synthesis.
- Chemical scaffold core: Consists of amino acid pieces connected by ester or amide bonds to form circular or straight structures [44].
- Advantageous pharmacophore characteristics: High amphiphilic qualities, structural stiffness, and hydrogen bonding capability [45].
- Relevance to therapy: It is used in immunosuppressive and antibacterial treatments, such as cyclosporine and vancomycin [46].
- Principal restrictions: They have significant manufacturing costs, limited oral bioavailability, and enzymatic breakdown.

The natural-product space also includes emerging food-derived bioactive peptides with defined molecular activity. For example, novel antioxidant peptides identified from tiger nut were shown to possess mechanistically interpretable redox activity, reinforcing the view that peptide-based natural products are not merely nutritional molecules but also tractable pharmacological entities with therapeutic potential [47].

### 2.8. Steroids and Saponins

- Origin of biosynthesis: Derived from the mevalonate pathway through triterpenoid biosynthetic intermediates.
- Chemical scaffold core: Characterized by a nucleus of cyclopentanoperhydrophenanthrene [48].
- Advantageous pharmacophore characteristics: Hydrophobic core with functional alterations like glycosylation and hydroxylation.
- Relevance to therapy: They play a role in membrane contacts, anti-inflammatory activities, and hormone control [49].
- Principal restrictions: Linked to toxicity, metabolic instability, and off-target hormonal effects.

## 3. Natural Products in Major Therapeutic Areas

The structural diversity, multi-target activity, and evolutionary optimization of the biological interactions of natural products have had an immense impact on modern pharmacotherapy in various fields of therapy. Their clinical performance, however, differs widely due to variations in molecular mechanisms, pharmacokinetic activities, and translational abilities [50]. This part critically analyzes natural products in key disease domains, assessing structure–activity relationships (SAR), molecular targets, efficacy, and clinical status, and also acts as a guide to the most serious limitations of natural products, including toxicity, resistance, and bioavailability [51]. Represented natural products and their therapeutic use is summarized in Table 2 [52,53].

Notably, the evidence for these compounds exists among clinically approved drugs (e.g., paclitaxel, digoxin), clinical trial candidates (e.g., curcumin), and preclinical leads, highlighting the disconnect between biochemical activity and effective clinical translation.

### 3.1. Anticancer Agents

Natural products have made a very significant contribution to oncology, especially by modulation of microtubule dynamics, topoisomerase inhibition, DNA intercalation and apoptosis induction [54]. Prominent examples are paclitaxel, vincristine, camptothecin and doxorubicin. From a SAR perspective, the taxane ring and C13 side chain in paclitaxel are crucial for microtubule stabilization, while the indole–indoles dimeric framework of vinca alkaloids allows tube binding and polymerization inhibition [55]. Camptothecin derives (using lactone E-ring integrity) to become a Topo I inhibitor, and anthracyclines (using a planar aromatic system) to become DNA intercalator (doxorubicin) [56]. Clinically, semisynthetic analogs like docetaxel and etoposide have improved pharmacological and toxicological statements, which underscores the value of chemical modification of natural scaffolds.

Beyond classical mechanisms such as microtubule disruption, topoisomerase inhibition, and DNA intercalation, natural product-derived agents can also target metabolic vulnerabilities in cancer. In this context, cannabinoids have been discussed as modulators of cancer-associated metabolic reprogramming, further supporting the concept that natural scaffolds may interfere not only with proliferative signaling but also with the metabolic plasticity that sustains tumor progression [57].

### 3.2. Antimicrobial and Antiviral Agents

Natural products form one of the pillars of anti-infective treatment. Antibiotics such as penicillin, streptomycin and erythromycin mainly function by inhibiting biosynthesis of the cell wall, the ribosomal function and protein synthesis respectively [58]. Key SAR features are the presence of the β-lactam ring strain, which is important for acylation of transpeptidases, aminoglycoside amino sugar residues important for binding to the ribosome, and macrocyclic lactone rings important for interactions with bacterial ribosomes [59]. In antiviral therapy, terpenoid and phenolic natural products—like artemisinin, glycyrrhizin—are used, for the generation of inhibition of viral replication and host–virus interactions [60]. Structural modifications that have increased stability and specificity for the target continue to increase their therapeutic scope.

### 3.3. Anti-Inflammatory Agents and Immunomodulatory Agents

Polyphenols and terpenoids both have strong anti-inflammatory properties through the regulation of NF-κB, COX, LOX and cytokines pathways [61]. Some of the notable compounds are curcumin, resveratrol, and boswellic acid. SAR analyses show that the phenolic hydroxyl groups, conjugated diketone systems, and hydrophobic terpenoid skeletons are important for the enzyme inhibition and redox modulation [62]. These compounds are increasingly studied as multi-target anti-inflammatory compounds, which have less adverse effects than synthetic NSAIDs [63].

### 3.4. Cardiovascular Therapeutics

Cardiac glycosides such as digoxin and digitoxin exert positive inotropic effects by inhibiting Na^+^/K^+^/ATPase activity, thereby improving cardiac contractility in heart failure and arrhythmias [64]. This mechanism causes a rise in intracellular calcium and a strengthening of the heart muscle. In addition, polyketides derivative statins (lovastatin) interfere with the biosynthesis of cholesterol [65]. The lactone ring and the dihydroxy acid pharmacophore are critical to enzyme binding and enzyme inhibition.

### 3.5. Neuroprotective and CNS-Active Natural Product

Alkaloids and flavonoids possess varied central nervous system (CNS) actions such as neuroprotective, cognition-enhancing and neurotransmitter-modulating effects [66]. Representative of molecules include galantamine, caffeine and quercetin. The tertiary amine functionality present in galantamine is responsible for cholinesterase binding; whereas, planar aromatic systems present in flavonoids are responsible for antioxidant and neuroprotective functions [67]. These scaffolds are becoming a rapidly growing area for Alzheimer’s disease and neurodegenerative diseases.

Recent work has also shown that mechanistic interpretation of neuroactive herbal products should extend beyond the parent compound to include biotransformation products. Using Astragaloside IV as an example in intracerebral hemorrhage, Hu et al. integrated microbial and hepatic biotransformation with network pharmacology, illustrating a more realistic framework for understanding how natural products exert CNS-directed effects in vivo [68].

### 3.6. Antidiabetic Agents and Metabolic Disorder Agents

Natural products with enzyme inhibitory and metabolic regulatory properties have shown promise in the management of diabetes [69]. Compounds found in berberine, ginsenoside Rb1 and epigallocatechin gallate regulate the signaling of the protein AMP, activated protein kinase (AMPK), glucose uptake, and insulin sensitivity [70]. SAR studies indicate the importance of cationic nitrogen centers, phenolic hydroxyl, and glycosidic links in metabolism. These compounds also play a role in obesity, dyslipidemia and metabolic syndrome [71].

## 4. Structure–Activity Relationships (SAR) of Bioactive Natural Products

Natural products’ molecular structure, distribution of functional groups, stereochemistry, and conformational characteristics all play a crucial role in determining their pharmacological effectiveness [72]. Structure–activity relationship (SAR) studies provide a methodical framework for comprehending how certain structural characteristics affect target binding, potency, selectivity, pharmacokinetics, and toxicity [73]. Natural compounds, in contrast to synthesized small molecules, often have rich functionalization and great stereochemical complexity, which makes SAR interpretation difficult but very instructive. This section concentrates on mechanistically significant SAR findings, highlighting the ways in which specific structural alterations result in adjustments to biological activity and therapeutic efficacy.

### 4.1. Functional Groups That Determine Bioactivity

Functional groups play a key role in how natural products are recognized, how strongly they bind, and how they react [74,75,76].

- Hydroxyl Groups (-OH): Often found in polyphenols like quercetin and epigallocatechin gallate, they positively affect hydrogen bond formation and enhance antioxidant activity through free radical scavenging and metal chelation, while also effectively chelating metals. The increase in hydroxylation can be associated with an increase in antioxidant capacity but a decrease in membrane permeability.
- Carbonyl and Lactones Moieties: In the case of camptothecin, the α-hydroxy-δ-lactone ring is involved in the stabilization of the drug–enzyme–DNA tertiary complex. Hydrolysis or ring opening has an appreciable negative effect on activity.
- Amine Functionalities: Alkaloids such as morphine are based on the fact that the key of their action is that they contain protonatable tertiary amines, which in turn interact ionically with receptor residues.
- Epoxide and Peroxide Bridges: The endoperoxide linkage in artemisinin is important in the generation of active oxygen species in the parasitic cell.
- Substituent Mapping Insight: Usually electron donating groups (-OH, -OCH_3_) will enhance the radical scavenging and enzyme inhibitory actions, whereas electronegative groups may increase the metabolic stability of the drug and drastically increase the target selectivity of the drug molecule.

### 4.2. Stereochemistry and Conformational Effect

Natural products contain a high number of chiral centers and rigid conformations that can have a pronounced effect upon binding orientation and receptor selectivity [77,78,79].

- In paclitaxel, the arrangement of atoms in the C13 side chain is important for how it connects to tubulin and helps stabilize microtubules.
- For vincristine, the dimeric indole–indoline structure and its specific arrangement are what allow it to interact with β-tubulin.
- Flavonoids exhibit planar conformations facilitating p–p stacking interactions with nutrient nucleic acids and proteins; flexible glycosides have conformational adaptability of membrane permeability.
- Conformational rigidity vs. flexibility: Rigid scaffolds will be more exclusive to binding sites while the flexible substituents will be more adaptable/increase pharmacokinetics.

### 4.3. Pharmacophore Mapping of Natural Products

Pharmacophore modeling is used to identify essential structural features necessary for biological activity in order to rationally design analogs as summarized in Table 3 [80,81,82].

### 4.4. Optimization of SAR

The following well-defined examples are intended to illustrate specific relationships between structure and function.

#### 4.4.1. Taxane Derivatives

Modification of paclitaxel gave docetaxel; the modification at the C10 and C13 substituents led to improved aqueous solubility and receptor affinity thereby increasing clinical efficacy [83].

#### 4.4.2. Podophyllotoxin Analogs

The etoposide precursor was the natural lignan podophyllotoxin, in which glycosidic modification and ring substitution have converted a tubulin inhibitor into a topoisomerase II inhibitor, offering an example of the critical importance of slight structural modifications on mechanisms of action [84].

#### 4.4.3. Curcumin Analogs

Sentence structure alteration of curcumin that focuses on [85] (a) stabilization of the b-diketone moiety and (b) substitution on aromatic rings has leapfrogged to providing improved (more bioavailable, metabolic stable and anti-inflammatory) analogs [86].

#### 4.4.4. Statin Optimization

The natural product lovastatin led to the discovery of synthetic statins in which modifications to the side chain and ring substitutions improved binding affinity to HMG-CoA reductase and improved lipid-lowering efficacy [87,88].

### 4.5. SAR Trends and Design Principles

Across the classes of natural products, several general principles of SAR are observed [89]:

- The hydrogen-bond donors/acceptors improve the binding with the target.
- For a compound to be permeable by membranes, lipophilicity balance (logP) is crucial.
- Rigid core + flexible side chain— a combination of both often produces best activity.
- Glycosylation, which is the process of adding sugar molecules to proteins or lipids, will increase solubility and may decrease permeability.
- Bio-isosteric replacement can be used to increase metabolic stability without loss of activity.

The SAR of the natural products indicates that biological activity is highly complex depending on the composition of functional groups, stereochemistry, and the topology of molecules [90]. Understanding these relationships makes it possible to optimize rationally, to design pharmacophores for rational drug design and to develop semisynthetic analogs with better therapeutic performance characteristics [91]. Consequently, SAR analysis is one of the pillars of establishing natural products into clinically viable drugs.

## 5. Chemical Modification Semisynthetic Derivatives

The natural products frequently have a strong biological activity, but their direct clinical usage is often limited due to suboptimal physicochemical and pharmacokinetic characteristics, such as low solubility, low metabolic stability, low bioavailability, and even toxicity [92]. Chemical modification and semisynthetic derivatization have emerged as key approaches to medicinal chemistry to overcome these obstacles, and to allow bioactive natural scaffolds to be converted into therapeutically useful products.

In contrast to SAR analysis, where the structure–activity relationships are studied, this section is more practical, as the chemical modifications are discussed to enhance drug-like properties without interfering with the pharmacophoric ones.

### 5.1. Strategies for Structural Optimization

Systematic optimization of functional groups and substituents in natural products is known as structural optimization, which aims to improve the biological activity and pharmacokinetics of these products. The process is usually initiated by identification of the pharmacophoric core, which is then followed by targeted alteration of the peripheral areas to enhance solubility, lipophilicity, and metabolic stability [93]. The classical example is the optimization of paclitaxel in which alterations changing the C10 and C13 positions resulted in the introduction of docetaxel [94].

These modifications enhanced solubility in aqueous solution, increased affinity of microtubules-binding and led to improvements in clinical efficacy. The same methods have been applied to various classes of natural products, where small variations in substituent electronic properties or steric effects significantly influence receptor interactions and off-target effects [95]. Optimization of structure needs to balance between enhancement of potency and preservation of preferable pharmacokinetics since too much modification may cause essential pharmacophore characteristics and loss of biological activity [96].

### 5.2. Prodrug Design from Natural Scaffolds

Prodrug strategies are extensively used to overcome pharmacokinetic issues of natural products, especially poor bioavailability, lack of solubility, and quick metabolism. In this approach, a bio reversible functional group is introduced which produces an inactive or less active derivative which can be cleaved in vivo to produce the active parent compound [97]. Natural product-derived prodrugs often involve esterification formation, carbamates or glycosides to increase membrane permeability and systemic absorption [98].

For example, a number of camptothecin analogs have been made as prodrugs in attempts to stabilize the lactone pharmacophore and enhance the plasma stability [99]. Similarly, natural phenolic products, such as curcumin, are modified using ester or ether derivatives, which have been found to improve bioavailability, enhance metabolic resistance, and maintain anti-inflammatory activity.

Current prodrug design is increasingly moving beyond simple masking strategies toward architectures that also control supramolecular behavior. Feng et al. showed that precise tailoring of doxorubicin prodrugs can enable stable nano assembly together with rapid activation and potent antitumor activity, highlighting how linker design and molecular packing can be co-optimized to improve therapeutic performance [100].

### 5.3. Synthetic Analogs Inspired by Natural Products

Natural products are often used as templates in the design of entirely synthetic analogs, enabling the retention of important pharmacophoric groups whilst simplifying the complexity of the molecule and enhancing drug-like properties [101]. This method is especially useful when natural compounds cannot be easily isolated, synthesized or scaled to be produced in large quantities [102].

The transformation of the natural product podophyllotoxin to etoposide serves as a clear example. The parent compound was altered structurally to turn it into a topoisomerase II inhibitor instead of a tubulin inhibitor and thus acquired better therapeutic applicability. On the same note, lovastatin optimization resulted in the creation of synthetic statins that were more potent, selective and had better pharmacokinetic characteristics.

These are some examples of how natural scaffolds can inform the design of simplified and more accessible drug molecules in a way that does not affect biological activity [103].

### 5.4. Improving Potency, Selectivity, and Stability

The main goal of semisynthetic modification is to enhance the performance of therapeutics by increasing the binding affinity, target selectivity, and metabolic stability. This is done by introducing substituents that enhance intermolecular interaction like hydrogen bonding, hydrophobic contacts and π–π stacking [104].

As an example, the metabolic stability and membrane permeability of flavonoids can be enhanced by methylation or glycosylation of the hydroxyl groups, but at the expense of antioxidant activity in some cases [105]. Derivatization in terpenoids like artemisinin has resulted in analogs with better pharmacokinetic properties and antimalarial effectiveness [106]. Structural changes should be tightly regulated in cardiac glycosides like digoxin, as they have a very narrow therapeutic index, and balancing efficacy and safety is important to prevent potential toxicity and ensure therapeutic effectiveness in treating conditions such as heart failure and arrhythmias [107].

Chemical modification and semisynthetic derivatization are important approaches in the development of natural products into contemporary substances for therapeutic use. Table 4 [108,109] lists representative semisynthetic derivatives and their pharmacological advantages. Therefore, optimization strategies should take into account the trade-offs of potency, selectivity and toxicity that are essential to clinical success [110].

## 6. Mechanisms of Action at Molecular and Cellular Levels

Natural products exert their pharmacological effects through well-orchestrated interactions with biomolecular targets such as enzymes, receptors, ion channels, nucleic acids and signaling proteins [111]. Owing to their structural diversity and stereochemical complexity, these compounds frequently interact with multiple targets at the same time and hence exert poly-pharmacological effects, which are especially beneficial in the treatment of multifactorial diseases [112]. Understanding the underlying molecular and cellular mechanisms of action of natural products is therefore imperative for rational drug design, optimization, and, ultimately, clinical translation.

### 6.1. Enzyme Inhibition

A large proportion of natural products act as enzyme inhibitors, through competitive and non-competitive or mechanism-based inhibition. The structural complementarity between natural products and enzyme active sites means that high affinity binding and selective inhibition is possible [113]. For example, lovastatin is a competitive inhibitor of the enzyme HMG-CoA reductase, that resembles the tetrahedral intermediate of the enzymatic reaction, and in turn, inhibits cholesterol biosynthesis [114].

Similarly, the alkaloid galantamine is used to inhibit acetylcholinesterase due to its action at the active site and peripheral anionic site of acetylcholinesterase, and this inhibits the breakdown of acetylcholine, thus improving the cholinergic neurotransmission used in neurodegenerative disorders [115]. Polyphenolic compounds such as epigallocatechin gallate act as several inhibitors against a variety of enzymes, including kinases and oxidoreductases, with hydrogen bonding and π–π interactions with catalytic residues. The existence of hydroxyl-rich aromatic systems is critical to such interactions.

### 6.2. Receptor Modulation

Natural products are often agonist, antagonist and allosteric regulators of cell receptors. Their three-dimensional shape and the orientation of the functional groups allow them to mimic the action of the endogenous ligand and the activation of the receptor [116]. The opioid alkaloid morphine works as an m-opioid receptor agonist, where parts of the molecule, like the protonated amine and phenolic hydroxyl group, interact significantly within the receptor’s binding area [117]. In contrast, flavonoids like quercetin modulate estrogen receptors, G-protein coupled receptors (GPCRs), and nuclear receptors, and are frequently partial agonists/modulators [118]. Steroidal natural products, such as cardiac glycosides such as digoxin, have indirect receptor-mediated effects through interference with the ion homeostasis inside each cell, ultimately leading to disruption of cardiac contractility and the signaling cascades.

### 6.3. Modulation of Signal Transduction Pathways

Many natural products work by changing the signaling networks inside cells that are involved in processes like cell growth, cell death, inflammation, and stress from free radicals [119]. The inhibition of the kinase, modulation of transcription factors, and redox regulation frequently mediate these effects. For example, curcumin blocks the activation of NF-κB which in turn decreases the expression of pro-inflammatory cytokines and enzymes (COX-2 and iNOS) [120]. Similarly, resveratrol modulates the proteins of the morphologically different signaling pathways (Sirt -1, AMP kinase, mitogen-activated kinase (MAPK), etc.) [121].

Mechanistic dissection of complex herbal systems is increasingly pathway-resolved. Recent studies have demonstrated how multi-component systems can be deconvoluted into constituent-level and pathway-level pharmacology, providing a more precise understanding of biological activity, which can lead to improved therapeutic strategies and targeted treatments in herbal medicine [122].

### 6.4. Interaction with Nucleic Acids and the Epigenetic Targets

Certain products of natural origin are specific molecular entities that can directly interact with DNA and chromatin organization and affect gene expression and epigenetic control. These interactions include DNA intercalation, inhibition of topoisomerase, and histone modification [123]. Anthracycline antibiotics like doxorubicin intercalate with DNA base pairs and block the enzyme topoisomerase II causing discontinuities in the DNA strand and apoptosis in the rapidly growing cells [124]. Alkaloids such as camptothecine stabilize the DNA–topoisomerase I cleavage complex which prevents the DNA from being relegated [125]. In addition, polyphenols such as resveratrol and flavonoids have been shown to affect epigenetic enzymes, such as histone deacetylases (HDACs) and DNA methyltransferases; their action affects gene expression patterns implicated in the onset of disease states.

Natural products have their pharmacological effects through many different and highly specific molecular mechanisms such as inhibition of enzymes, modulation of receptors, interference of signal transductions, and nucleic acid interaction [126]. Their complexity in structure and diversity in functional group composition allows, therefore, target multiplicity, which is especially beneficial in the treatment of complex and multifactorial diseases [127]. A complete knowledge of these mechanisms not only will advance the goal of rational drug design and optimization, but will assist in addressing the development of future generations of therapeutics that use natural scaffolds.

## 7. Pharmacokinetics and ADMET Considerations

The successful occurrence of natural products into clinically effective therapeutics requires an orderly grasp of the pharmacokinetic behavior and ADMET (Absorption, Distribution, Metabolism, Excretion and Toxicity) properties [128]. Despite their high biological activity, natural products often face challenges such as limited bioavailability, rapid metabolism, poor solubility, and off-target toxicity [129]. Table 5 [130,131,132] summarizes the characteristics of pharmacokinetics and ADMET. Consequently, optimization of ADMET characteristics by medicinal chemistry surgical strategies, formulation strategies and the prodrug design is therefore crucial to bring out their full pharmacotherapeutic potential.

### 7.1. Absorption and Bioavailability

The oral absorption of natural products is influenced by physicochemical data such as lipophilicity (log P), molecular weight, capacity for hydrogen bonding and polarity [133]. Highly polar compounds, which may include polyphenols like curcumin and epigallocatechin gallate, exhibit poor intestinal permeability and low systemic bioavailability due to extensive first-pass metabolism and efflux through transporters such as P-glycoproteins [134]. Conversely, lipophilic terpenoids such as paclitaxel have poor water solubility and can only be delivered orally; they need to be formulated using parenteral routes or formulations with nanoparticles [135]. Structural modifications such as esterification, glycosylation or prodrug formation are widely used in increasing membrane permeability and absorption efficiency.

### 7.2. Distribution and Plasma Protein Binding

After absorption, natural products are carried throughout the body’s tissues based on their lipophilicity, affinity with plasma proteins and molecular size [136]. Highly lipophilic molecules such as doxorubicin and paclitaxel have high binding affinity to plasma proteins (e.g., albumin), as well as preferential accumulation in lipid-rich tissues [137]. The distribution profile also has an effect on blood–brain barrier (BBB) permeation which is critical for CNS active natural products such as galantamine [138]. Structural properties like relief of polarity, molecular rigidity, and proper hydrogen bond donor/acceptor balance help in CNS penetration.

### 7.3. Metabolism: Phase I and Phase II Biotransformation

Natural products undergo a wide range of metabolism by hepatic cytochrome P450 enzymes (Phase I), and by conjugation reactions (Phase II), such as glucuronidation, sulfation, and methylation [139]. These metabolic processes can cause the compounds of natural products to be activated, deactivated, or experience toxification [140]. For example, resveratrol is quickly converted to glucuronide and sulfate conjugates, which limits its systemic exposure despite its potent in vitro activity. Similarly, morphine is glucuronidases and produces both active and inactive glucuronidases metabolites, all of which affect morphine analgesia and toxicity [141]. Strategic chemical modification (e.g., by blocking metabolically labile sites or by introducing steric hindrance) can be used to increase metabolic stability and increase systemic circulation.

### 7.4. Toxicity and Safety Profiles

Although natural products are generally thought to be safe, because they may be more likely to be, many have dose-dependent toxicity and narrow therapeutic indices [142]. For example, digoxin exhibits strong cardiotonic action but needs severe attention to the dose as a result of risk of arrhythmias and toxicity [143]. Cytotoxic natural products such as doxorubicin have been linked to cardiotoxicity and oxidative stress and therefore need clinical management. Toxicity can be caused by reactive functional groups, metabolic activation of electrophilic intermediates, or off-target interactions [144], which can lead to adverse effects such as organ damage or impaired drug efficacy if not properly managed. Modern toxicological evaluation combines in vitro screening, in vivo studies and computational toxicity prediction models to ensure safety in the drug development process.

### 7.5. Drug–Herb Interactions

Natural products can have an important impact on the pharmacokinetics of co-administered medications through inhibition or induction of enzymes; modulation of transporters; and displacement binding of proteins [145]. For example, polyphenolic compounds like quercetin may inhibit the CYP3A4 and P-glycoprotein thus affecting plasma concentrations of co-administered pharmaceuticals. Similarly, terpenoids and alkaloids may alter drug-metabolizing enzymes resulting in clinically relevant drug interactions [146]. These interactions especially occur in patients taking herbs as supplements to other conventional drugs, and more globally in polypharmacy and chronic disease management [147]. Pharmacokinetic evaluation and ADMET considerations are an important determinant of the clinical success for natural product-based therapeutics. Despite having powerful biological activities, issues in their processing, such as deficiencies in bioavailability, a rapid metabolic microenvironment, and toxicity, necessitate the need to perform rational optimization through medicinal chemistry and advanced formulation strategies [147]. Thorough knowledge of ADMET profiles not only guarantees the development and approval by the responsible state bodies for a drug, but also the safe and effective use of natural products in contemporary pharmacotherapy.

Predictive computational models are increasingly important for anticipating interaction risks. Advanced frameworks incorporating stereochemical information have shown improved accuracy in predicting drug–drug interactions and may be extended to herb–drug interaction assessment [148].

## 8. Advanced Drug Delivery Systems for Natural Products

**Figure 3:**
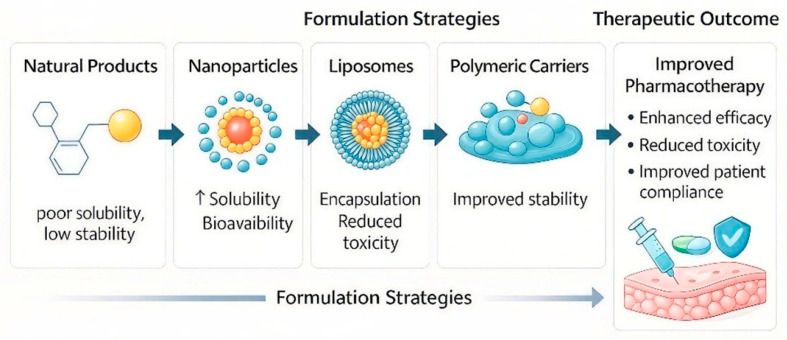
Advanced natural product drug delivery systems and therapeutic results.

The use of natural products in therapy is often hindered by undesirable physicochemical and pharmacokinetic characteristics, such as a low aqueous solubility, low bioavailability, high metabolism, and instability at physiological conditions [149]. Advanced drug delivery systems are the most important enabling technologies to address these limitations [150]. Nevertheless, despite the many formulation strategies suggested, their clinical implementation has been fragmented, and a more critical approach is required to understand the effectiveness, scalability, and regulatory feasibility of these strategies [151]. The bioavailability and therapeutic effectiveness of natural substances are enhanced by advanced formulation techniques such polymeric carriers, liposomes, and nanoparticles, as shown in Figure 3.

Emerging delivery strategies such as metal-coordination-based systems provide additional control over drug loading, protection, and release behavior, representing a promising future direction for natural product therapeutics.

### 8.1. Nanoparticle-Based Delivery Systems

Nanoparticle-based formulations have come up as a dominant platform for enhancing the biopharmaceutical properties of hydrophobic natural products [152]. Polymeric nanoparticles and solid lipid nanoparticles and nanocrystals facilitate controlled drug release, improving their permeability and extending their system circulation [153].

For example, nano formulations of paclitaxel have led to notable enhancements in the aqueous dispersibility and tumor targeting capabilities of paclitaxel, and have reduced the needed addition of toxic solubilizing agents [154]. Similarly, curcumin encapsulation into polymeric nanoparticles increases the chemical stability and cell uptake of curcumin, thereby enhancing its anti-inflammatory and anticancer activity [155]. Nanoparticle modification of surface with ligands or antibodies allows for the active targeting of diseased tissue, especially in oncology, where enhanced permeability and retention effect (EPR) provide features to be accumulated selectively in tumor microenvironments.

Carrier-free nanomedicine is emerging as an attractive strategy due to high drug loading and reduced reliance on excipients. Such systems have demonstrated enhanced therapeutic efficacy while minimizing toxicity in preclinical models. Despite promising laboratory results, many nanotechnology-based systems fail to reach clinical translation due to challenges in large-scale manufacturing, stability, regulatory approval, and high production costs [156].

### 8.2. Liposomes and Phytosomes

Lipid-based carriers like liposomes and phytosomes are especially excellent candidates for the delivery of lipophilic or amphiphilic natural products [157]. Liposomes are made of phospholipid bilayers containing both hydrophilic and hydrophobic drugs to enhance solubility and systemic circulation time. Clinically, liposomal preparations of doxorubicin have been shown to have decreased cardiotoxicity, as well as an increased therapeutic index [158]. Phytosomes, complexation of phytochemicals with phospholipids, have been widely used in the improvement of the bioavailability of polyphenols like resveratrol and flavonoids like quercetin [159]. These systems enhance membrane permeability, gastrointestinal absorption and metabolic stability, and are therefore especially suitable for oral administration of compounds derived from plants.

### 8.3. Polymeric and Targeted Delivery Systems

Polymeric delivery systems involve the use of biodegradable polymers, namely PLGA, chitosan and PEG-based materials to prepare controlled-release formulations of natural products [160]. These systems offer the possibility of sustained release of the drug, lower dose frequency and better therapeutic compliance [161]. Targeted polymeric carriers can be designed to react to changes in pH, redox status, and enzymes and allow for the site-specific release of drugs.

For example, polymer-drug conjugates of camptothecin improve the stability of lactone rings and tumor targeting and therefore improve anticancer activity [162]. Additionally, conjugation of natural products with antibodies, peptides or receptor ligands are being used to facilitate precision delivery to a specific cell type for better selectivity and reduced off-target toxicity [163].

### 8.4. Strategies for Enhancing Solubility and Stability

Enhancing solubility and chemical stability is crucial for many of the natural products that have undesirable compatibility with water or quickly suffer degradation. Strategies include solid dispersions, cyclodextrin inclusion complexes, micellar systems, and prodrug formation among others [164]. For example, cyclodextrin complexation of artemisinin has improved its aqueous solubility and stability and should facilitate oral and parenteral administration. Similarly, micellar formulations of hydrophobic terpenoids and flavonoids improve dissolution rate as well as intestinal absorption [165]. Chemical stabilization by esterification, glycosylation or encapsulation in protective matrices may be achieved to prevent oxidative degradation and hydrolysis with a longer shelf life and, hence, an extended therapeutic activity [165].

Advanced drug delivery systems are inevitable in overcoming the pharmacokinetic and physicochemical limitations of natural products [166]. Nanotechnology, lipid carriers, polymeric systems, and targeted delivery strategies can significantly improve the solubility, stability, bioavailability, and therapeutic index of natural product-derived drugs [167]. These innovations help fill the gap between powerful natural bioactivity and clinical viability so that structurally complex natural compounds can now be effectively converted into safe and efficacious pharmacotherapies.

## 9. Analytical Techniques and Structure Elucidation

The structural characterization, purity evaluation and quantitative analysis of natural products lie at the core of pharmaceutical chemistry-driven drug discovery [168]. The structural complexity and stereochemical diversity of natural products and closely related analogs create a need for a multidimensional analytical framework to establish unequivocal structural identity and support the SAR-based optimization of a natural product [168]. This section describes the current state-of-the-art chromatographic, spectroscopic, and hyphenated analytical tools used in natural product research and development.

### 9.1. Chromatographic Techniques for Isolation and Purification

#### 9.1.1. Thin-Layer Chromatography (TLC) and High-Performance Thin-Layer Chromatography (HPTLC)

TLC (thin-layer chromatography) and HPTLC (high-performance thin-layer chromatography) are still very useful when there is a need for quick qualitative profiling and dereplication of a natural extract [169]. The combination of several solvent systems and derivatization reagents allows for the visualization of various classes of metabolites (e.g., phenolics (NP/PEG reagents), alkaloids (Dragendorff’s reagent) and terpenoids (anisaldehyde-sulfuric acid)) [170]. HPTLC offers a better resolution and densitometric quantification to ease comparative phytochemical fingerprinting among plant species and plant batches. Such techniques are especially useful in early-stage workflow to provide direction to isolate key scaffolds, e.g., curcumin, berberine and quercetin [171].

#### 9.1.2. High-Performance Liquid Chromatography (HPLC) and UHPLC

The main method for the purification and quantitative analyses of natural products is prepared by using reversed phase high-performance liquid chromatography (RP-HPLC) [172]. Advanced stationary phases (C18, phenyl-hexyl, biphenyl) allow the separation of compounds with structurally similar compounds by hydrophobic interaction forces and π–π forces [173,174]. Ultra-high-performance liquid chromatography (UHPLC) provides even higher resolution, sensitivity, and throughput, which are required for complicated matrices in which several stereoisomers and glycosylated derivatives are present [175]. The gradient elution strategies are frequently optimized to resolve natural product classes like flavonoids, alkaloids and terpenoids.

#### 9.1.3. Gas Chromatography (GC) and GC–MS

Gas chromatography is especially suitable for volatile and semi-volatile natural products such as components of essential oils, such as monoterpenes and sesquiterpenes [176]. Gas chromatography coupled with mass spectrometry (GC-MS) allows library-based spectral matching and thus rapid dereplication and identification, e.g., of limonene and eugenol [177]. Derivatization methods (e.g., sialylation) are commonly used to enhance the volatility and the thermal stability of hydroxylated natural products before GC analysis [178].

### 9.2. Spectroscopic Techniques for Structural Elucidation

#### 9.2.1. Nuclear Magnetic Resonance (NMR) Spectroscopy

NMR spectroscopy is the mainstay of natural product structure determination; thus, NMR provides information for molecular connectivity, stereochemistry and conformational dynamics [179,180].

- 1D NMR (one-dimensional nuclear magnetic resonance) for both ^1^H (proton) and ^13^C (carbon-13) shows the chemical environments, multiplicity, and coupling constants.
- 2D NMR techniques (COSY, HSQC, HMBC, NOESY/ROESY) prove the proton–proton and proton–carbon correlations and fully solve the structure, even for very complex molecules [181]. For example, the determination of the complete stereochemistry in polycyclic molecules like artemisinin and paclitaxel has been made with the help of advanced multidimensional NMR technique.

#### 9.2.2. Mass Spectrometry (MS) and Tandem MS (MS/MS)

Mass spectrometry is useful for offering precise molecular mass and elemental composition and fragmentation patterns and is essential for the identification of unknown natural products and determination of molecular formulas [182]. High resolution mass spectrometers (HRMS) and time-of-flight (TOF) mass spectrometers allow us to obtain accurate mass results; whereas tandem mass spectrometry (MS/MS) provides details on structures from diagnostic fragment ions, especially suitable for glycosides and conjugated metabolites [183]. For example, fragmentation analysis is routinely applied to characterize such molecules as the anthracycline class of antibiotics like doxorubicin or the macrolide lactone like erythromycin [184].

#### 9.2.3. Infrared (IR) and UV–Visible Spectroscopy

Infrared spectroscopy is used to determine functional groups, such as hydroxyl, carbonyl, amine, and aromatic functional groups, by a characteristic vibrational frequency [185]. UV-visible spectroscopy informs conjugated p-electron systems, particularly in polyphenols, flavonoids, and quinones [186]. These techniques are used as complimentary tools to NMR and MS for quick confirmation of functional group composition of compounds such as resveratrol and curcuminoids.

### 9.3. Hyphenated and Advanced Analytical Techniques

#### 9.3.1. LC-MS/MS and LC-HRMS

Liquid chromatography coupled with tandem mass spectrometry (LC-MS/MS) is among the gold standards of metabolite profiling, quantitative bioanalysis and pharmacokinetic studies [187]. LC-HRMS enables the detection of untargeted metabolomics and trace-level constituents in complex biological matrices [188]. These platforms become important tools for the identification of bioactive metabolites, degradation products and conjugates of natural products in vivo.

#### 9.3.2. X-Ray Crystallography

Single-crystal X-ray diffraction provides definitive three-dimensional structural information, absolute configuration, stereochemistry, etc. [189]. It is especially useful for compounds of a rigid and crystal nature, such as morphine and colchicine, where stereochemical assignment is critical to biological activity, as incorrect stereochemistry can lead to ineffective or harmful biological effects [190].

#### 9.3.3. Techniques Manuscript CD, ORD, VCD (Chiroptical)

Chiroptical spectroscopic techniques, namely, circular dichroism (CD), optical rotatory dispersion (ORD), and vibrational circular dichroism (VCD) are being widely used to determine the absolute configuration of chiral natural products, particularly without any crystallographic data [191,192]. Of special use are these techniques for flexible molecules and glycosylated derivatives. for which such stereochemical assignment is difficult, as they provide valuable insights into the three-dimensional arrangement of atoms in these complex structures.

### 9.4. Quantitative Analysis and Standardization of Natural Products

Quantitative determination of bioactive constituents is essential in ensuring quality control, dose standardization and reproducibility in pharmacological studies [193]. Validated analytical methods (HPLC, LC-MS/MS) are used to determine the amount of marker compounds such as [194]:

- Epigallocatechin gallate in botanical extracts.
- Ginsenosides in the Panax species.
- Withaniferin A in *Withania somnifera.*

Method validation parameters (linearity, precision, accuracy, LOD/LOQ, robustness) are ensured to be in the context of ICH Q2(R2) guidelines, which guarantees regulatory compliance in pharma [195].

### 9.5. Dereplication, Metabolomics, and Chemo-Informatics Approaches

Modern natural product research combines dereplication approaches that make use of spectral databases (such as GNPS and METLIN) and chemo-informatics tools to identify known products fast and prioritize the new ones [196]. Metabolomics platforms that utilize LC-HRMS and NMR-based fingerprinting provide holistic profiling of phytochemical composition, which is correlated with biological activity through the use of multivariate statistical models, such as PCS-DA. These approaches help to speed up the discovery of leads, SAR mapping and mechanism of action studies in complex natural product systems [197].

Advanced analytical and spectroscopic methodologies are the pillars of natural product pharmaceutical chemistry as they give rise to the accurate solution of molecular architecture, stereochemistry and functional groups composition [198]. The amalgamation of chromatographic separation highresolution spectrometry and the computational analysis contribute to a robust framework aimed at structure-based drug discovery, the optimization of substructure and sustainability, screening (SAS) optimization and ensuring standardization of quality for the therapeutics obtained from a natural product [199]. These analytical advancements continue to create a bridge between the old-fashioned natural product research and the contemporary drug development pipelines so that structurally complex, natural scaffolds could be efficiently translated into clinically viable pharmacotherapeutic agents. Integrated molecular networking approaches are increasingly used to accelerate dereplication and prioritize novel compounds, reducing redundancy in natural product discovery workflows.

Recent progress in dereplication has also been driven by integrated molecular-networking workflows. For example, IMN4NPD was developed to improve natural-product dereplication by combining molecular-networking logic with faster recognition of known compounds, thereby helping reduce rediscovery and improving prioritization of chemically novel features during extract analysis [200].

## 10. Challenges and Barriers in Natural Product-Based Drug Development

Natural products remain in a central position in the field of drug discovery due to their special properties of structural diversity, stereochemical richness, and biological interaction optimization via evolution [201]. Despite their historical success and widespread use in pharmacotherapy, translating natural products into clinically viable drugs involves a series of scientific, technological, regulatory, and economic challenges [202]. Addressing these limitations needs a combination of innovative approaches in pharmaceutical chemistry, synthetic biology, and computation to achieve the full therapeutic potential of natural scaffolds [203], which may include developing new methods for drug formulation, enhancing bioavailability, and ensuring regulatory compliance [203].

### 10.1. Scientific and Chemical Challenges in Natural Product Research

One of the most significant challenges in natural products-based drug discovery is the inherent complexity of the structures of many bioactive molecules [204]. Compounds such as paclitaxel, vancomycin and artemisinin possess several stereo-centers, macrocyclic frameworks and sensitive functional groups, which have made the elucidation of their structures, chemical synthesis and optimization of SAR more challenging [205]. These structural features, though vital in determining biological activity, are often a limitation in terms of synthetic accessibility and scalability properties. A scientific drawback is that the predictor’s scope is limited to the low natural amounts of many bioactive compounds found in their native sources [206]. For example, the first extraction of paclitaxel from *Taxus brevifolia* bark produced only small amounts because of supply constraints in clinical development.

Similarly, compounds like camptothecin and podophyllotoxin have to undergo large-scale extraction and purification methods which are resource-intense and environmentally demanding [207]. Chemical instability and metabolic lability are also big problems. Functional groups such as lactones, epoxides and glycosidic linkages are susceptible to hydrolysis and enzymatic hydrolysis and consequently have short shelf stability and poor in vivo stability [208]. These problems require modification strategies for the structure to enhance stability without the loss of pharmacological activity.

### 10.2. Pharmacokinetic and Pharmacodynamic Limitations

From a pharmaceutical chemistry standpoint, many natural products have unfavorable pharmacokinetic properties, such as low aqueous solubility, poor membrane permeability, rapid metabolism and low oral bioavailability [209]. Polyphenolic compounds like curcumin and resveratrol are prototypical examples where a high level of first pass metabolism and rapid conjugation was observed to greatly restrict exposure in the systemic circulation [210]. In addition, such natural products display off-target effects as well as small therapeutic windows. For example, cardiotoxicity with anthracyclines like Doxorubicin poses the need for better selectivity and delivery [211]. Variations in pharmacodynamic responses caused by complex multi-target interactions further complicate dose optimization and clinical translation [212]. These are the pharmacokinetic and the pharmacodynamic limitations that emphasize the necessity of medicinal chemistry optimization, prodrug design, and advanced drug delivery systems aimed at optimizing drug-like properties [213,214].

### 10.3. Standardization, Quality Control, and Regulatory Barriers

The chemical heterogeneity of natural product extracts presents significant challenges in terms of standardization, reproducibility, and quality control. Variability in species of plants, geographical origin, harvesting conditions and extraction methodology result in batch-to-batch inconsistency of phytochemical composition [215]. The marker-based standardization approach using compounds like epigallocatechin gallate or withaferin A is often used, but this approach may not capture the full extent of synergistic effects of complex mixtures of phytochemicals, which can lead to inconsistencies in therapeutic outcomes and efficacy in clinical applications [216]. From the regulatory perspective, the natural products are classified as botanical drugs, dietary supplements or pharmaceutical agents depending on the jurisdiction, resulting in different ambiguity in the approval pathways [217]. The rigorous requirements around toxicological evaluation, clinical efficacy and manufacturing quality (GMP compliance) can be significant financial and logistic barriers for commercialization.

### 10.4. Sustainability, Biodiversity, and Ethical Considerations

The growing market demand for bioactive natural products entails critical concerns on sustainability, biodiversity conservation and ethical sources [218]. Overharvesting of medicinal plants and marine organisms jeopardize ecological and balance and long-term availability of valuable chemical entities [219]. For example, large-scale extraction of alkaloids such as vincristine and vinblastine from *Catharanthus roseus* has previously been very resource-demanding due to the large amount of biomass used; thus, there is a need to develop sustainable cultivation methods, plant cell cultures and biosynthetic production approaches [220]. Furthermore, due consideration of ethical issues associated with bioprospecting and benefit sharing with indigenous communities is gaining emphasis under international conventions such as the Convention on Biological Diversity (CBD) and the Nagoya Protocol [221].

### 10.5. Emerging Technologies and Future Directions

Despite these challenges, the future of natural product-based drug discovery is extremely bright because of the fast-progressing advancements in interdisciplinary technologies [222]. The combination of synthetic biology, metabolic engineering, and genome-mining is supporting the sustainable synthesis of natural products that are highly complex and its analogs from engineered microbial platforms [223]. Artificial intelligence (AI) and machine learning is reshaping natural product research through virtual screening, assisting in de novo design of scaffolds, and anticipating and progressively optimizing leads, as well as advantageously speeding up the identification of leads [224]. In parallel, network pharmacology and systems biology studies are forming new insights into the multi-target mechanisms of natural products in multi-targeted illnesses, for example, cancer, neurodegeneration, and metabolic disease [225]. The innovations in green chemistry and biocatalysis are also impacting the efficiency and sustainability of the environment toward natural product synthesis and derivatization, leading to reduced waste and lower energy consumption in the production processes [226].

Furthermore, nanotechnology-based delivery systems and targeted drug delivery platforms are tackling long-standing issues related to solubility, stability, and tissue specificity. Natural products continue to be an irreplaceable source of chemically disparate and biologically active starting materials for the development of contemporary drugs [227]. While there are still serious challenges such as chemical complexity, pharmacokinetics, standardization, and sustainability, continuous development in the field of pharmaceutical chemistry and other allied subjects is slowly working on overcoming these shortcomings [228]. The future of pharmacotherapy that is based on natural products will rely on the synergy between traditional knowledge and cutting-edge scientific innovation to rationally design, optimize, and ultimately clinically translocate natural product-derived therapeutics [229]. With an ongoing interdisciplinary partnership, natural products are set to be at the heart of expanding the development of next-generation precision medicine and global health innovation, particularly by integrating insights from both traditional practices and modern scientific research to address current health challenges and improve patient outcomes [230,231].

### 10.6. Supply and Sustainability Issues

One of the major hurdles for the natural product-based development of drug candidates is the spatial and temporal limitation and unsustainability of the supply of bioactive compounds from natural sources [232]. Many pharmacologically important molecules are present in low concentrations and thus require large-scale harvesting of plant or marine biomass [233]. Historically, compounds such as paclitaxel from Taxus plants and artemisinin from *Artemisia annua* are examples of how ecology and economics manage themselves under the form of large-scale extraction [234].

In addition to environmental concerns, geographical (and seasonal) variability can affect metabolite concentration, leading to inconsistent supply chains. Modern strategies like plant tissue culture, heterologous expression systems, and metabolic engineering are becoming more widely employed to provide sustainable production [235]. Furthermore, semisynthetic methods developed for the paclitaxel-related analogs represent viable solutions to deter the requirement for natural biomass sigma and yet preserve the structural integrity and biological activity, thereby ensuring a more reliable and sustainable supply of these important compounds.

### 10.7. Structural Complexity and Synthesis Barriers

Natural products can yield highly complex molecular structures, with a large number of stereo centers, macrocycles, spiro structures and labile functional groups [236]. This structural complexity (for example, vancomycin, erythromycin, etc.) represents substantial problems in total synthesis, chemical modification, and scalable manufacturing [237]. The synthesis of such molecules often includes several reaction steps yielding low overall yields and are therefore of only limited industrial feasibility [238]. Also, stereochemical control and protecting group strategies are sometimes factors that complicate route optimization, leading to increased costs and longer development times for the synthesis of these complex molecules. Advances in asymmetric catalysis, chemoenzymatic synthesis, and biocatalytic transformations are gradually solving these issues to allow more efficient access to complex scaffolds and their analogs for studies of structure–activity relationships (SARs) [239].

### 10.8. Standardization and Quality Control

The development of natural product-based pharmaceuticals requires the use of rigorous protocols for quality control and standardization to ensure the reproducibility, safety and efficacy of these products [240]. Natural extracts are complex and complex mixtures by nature, the cultivation and the extraction methods as well as storage can create considerable variability in composition [241].

Marker compound-based standardization based on molecules with well-known chemical structures such as epigallocatechin gallate and withaferin A are commonly used; however, such approaches may not be entirely sensitive to the synergistic pharmacological actions that are possible in multi-component systems [242]. Therefore, more and more modern quality control frameworks are based on chromatographic fingerprinting, metabolomic profiling, and chemometric analysis to obtain all-round characterization and batch-to-batch consistency.

### 10.9. Regulatory Challenges

The inconsistency in classification, safety evaluation requirements, and worldwide regulatory norms still complicates the regulatory approval of therapeutics derived from natural products [243]. Depending upon jurisdiction, natural products can be classified as botanical drugs, nutraceuticals or conventional pharmaceuticals, which have different regulation processes. Regulators require a coherent quality, well-defined composition, and reproducible pharmacological activity for acceptance [244].

Furthermore, issues such as the protection of IP, clinical trial design for multi-component systems, and compliance with Proper Manufacturing Practice (GMP) pose further challenges [245]. Harmonization of international regulatory systems and the design of science-based guidelines for botanical drug approval are determining factors in the spectacular push of natural product therapeutics into mainstream therapeutics, as they help ensure safety, efficacy, and quality in the development and approval processes.

## 11. Future Perspectives and Emerging Trends

The future of natural product-based drug discovery is being changed by technological convergence in pharmaceutical chemistry, systems biology and computational sciences [246]. Emerging strategies are making it possible to better discover, optimize, and clinically translate NPs and their derivatives [247].

### 11.1. Marine and Microbial Natural Products

Marine ecosystems and microbial communities are unexplored resources of structurally novel and biologically potent natural products [248]. The compounds Trabectedin and Salinosporamide A demonstrate the potential of marine-derived metabolites. Similarly, microbial sources, actinomycetes and endophytic fungi have provided clinically relevant agents such as Rapamycin and Ivermectin [249]. Advances in metagenomics and culture-independent techniques are making many microorganisms that were once uncultivable available and therefore new chemical diversity available [250].

### 11.2. Genome Mining and Synthetic Biology

Genome mining has become a revolutionary tool to discover cryptic biosynthetic gene clusters (BGCs) for biosynthesis of natural products [251]. The development of novel genetic engineering tools for exploring and exploiting new metabolite potentials through bioinformatic analysis and heterologous expression is now possible, enabling the discovery and synthesis of metabolites that had not been possible until now [252]. Synthetic biology offers the possibility of further engineering of biosynthetic pathways for improved yield, the production of structural analogs and novel functionalities [253]. These methods are really important for complex molecules like lovastatin and etoposide, as pathway engineering can enhance scalability and structural variation, which is crucial for their production for pharmaceutical applications and for meeting market demands.

### 11.3. Multi-Target and Network Pharmacology Approaches

Natural products have been shown to be poly-pharmacological in action, often targeting multiple molecular pathways and signaling mechanisms, unlike many small molecules developed in synthetic laboratories that are designed for a single target [254]. This property is especially useful when it comes to multifactorial diseases such as cancer, neurodegenerative disorders and metabolic syndromes [255]. Integration of omics data, protein–protein interaction networks and computational modeling, called network pharmacology, is used to understand the effects of natural products on a system-level. Compounds like curcumin and resveratrol are both examples of multi-target modulators that can regulate inflammation and oxidative stress, as well as cell survival pathways [256].

Computational prioritization frameworks can further strengthen network-based drug discovery from complex biological datasets. Although developed in a repurposing context, the indicator-regularized non-negative matrix factorization strategy reported by Tang et al. illustrates how association-learning models can be used to rank candidate therapeutics from sparse biological networks, an approach that could be adapted to natural-product repurposing and multi-target prioritization [257].

### 11.4. Personalized Medicine and Natural Products

Incorporating natural products into the structure of personalized medicine is a promising frontier in the area of pharmacotherapy [258]. Advances in pharmacogenomics, biomarkers, discoveries and also individual stratification of patients allow for personalized therapeutic approaches based on genetic and metabolic profile. Natural products with optimal safety profiles, one of the advantages of multi-target approaches, are a professional choice for use of personalized therapeutic regimen [259]. Moreover, the combination of natural product-derived agents with conventional drugs provides prospects for synergistic therapy, dose reduction, and the reduction in negative effects, which can enhance treatment efficacy and improve patient outcomes in personalized medicine.

## 12. Future Outlook

Despite notable advancements in the area of nanotechnology-based natural chemical delivery systems, there is still a dearth of successful applications of these systems from laboratory research to clinical and commercial healthcare. One of the main obstacles will also be the difficulty of growing manufacturing procedures. When scaling up to big production levels, nanocarriers that work well in the lab sometimes struggle to maintain stability, uniformity, and persistency. However, a successful technique applied at the nanoscale readily translates to huge scales, provided that the challenges of stability, uniformity, and persistency are adequately addressed during the scaling process.

Furthermore, safety concerns pertaining to nanomaterials present some very difficult obstacles. Regulatory clearance poses a challenge due to the incomplete understanding of long-term toxicity, bioaccumulation, immunogenicity, and environmental effects. The lack of established methods in the toxicity assessment exacerbates these worries, making it difficult for researchers and regulatory bodies to evaluate the safety of nanomaterials effectively.

Economic factors are also significant but, in addition to the fact that large-scale manufacturing is economically challenging, the output is expensive due to the high cost of raw materials, specialized equipment, and the fabrication process. This is particularly problematic when compared to the conventional formulas, which are easier to make and comparatively less expensive. Therefore, to facilitate their successful commercialization, future research should focus not only on improving effectiveness but also on developing scalable, affordable, and safe nanocarrier systems and uniform regulatory frameworks.

Beyond classical lipidic and polymeric systems, coordination-driven delivery is opening an additional design space for macromolecular therapeutics. Recent advances in metal ion-mediated macromolecular drug delivery indicate that metal coordination can be exploited to regulate loading, protection, responsiveness, and release behavior, offering a useful conceptual framework for next-generation delivery systems in natural-product and hybrid therapeutic formulations [260].

## 13. Conclusions

Natural products are still a singularly valuable source of structurally differentiated and biologically active substances used in pharmacotherapy today. This review has shown that the best way to understand their therapeutic use is through an integrated structure–function–translation model that links molecular structure to pharmacological activity, pharmacokinetic behavior, and clinical use. In various fields of therapy, natural products have shown great promise, but their transfer to clinical usefulness as drugs requires a complicated interaction between chemical structure, target affinity, and systemic activity, which must be carefully optimized through extensive research and development processes to ensure efficacy and safety in patients.

One important lesson that has come up in this analysis is that biological potency is not enough to guarantee clinical success. Numerous natural products have a high in vitro efficacy and do not translate because of constraints including low bioavailability and metabolic unsteadiness, toxicity, and inconsistency in composition. Conversely, there have been examples of successful agents in clinical use (paclitaxel, artemisinin, and digoxin) whose optimization of structure, semisynthetic alteration, and formulation have provided solutions to inherent shortcomings and made their use practical. Therefore, natural product-based drug discovery is not just in the identification of novel compounds but will be optimized methodically in the future through medicinal chemistry and translation clinical approaches.

Despite this progress, a number of unresolved issues persist. Structural complexity continues to limit accessibility to synthesis, large-scale synthesis, and pharmacokinetic and ADMET processes necessary for clinical development. Moreover, standardization, reproducibility, and quality control problems are also critical, especially in the case of plant-derived and multi-component systems, as they can lead to inconsistent results and hinder the approval process for new therapies. Sustainability issues and regulatory hurdles also make developing the pipeline complex, as they can lead to increased costs, longer development times, and potential delays in bringing new therapies to market. These challenges are going to need concerted efforts with synthesis chemistry, systems pharmacology, high-order analytics and regulatory science.

In the future, new technologies will provide some promising prospects to transform natural product research. Genome mining and synthetic biology are increasing access to chemical space that was previously inaccessible, and network pharmacology and computational models allow an understanding of multi-target mechanisms on a systems level. New drug delivery systems, such as nanotechnology and carrier-free systems, enhance bioavailability and target specificity. Meanwhile, predictive computational frameworks are also starting to improve prioritization of bioactive compounds and predict risks of interactions to aid in more efficient and rational drug development processes.

Future studies should thus focus on: (i) incorporation of SAR-driven design with pharmacokinetic optimization, (ii) establishment of scalable and sustainable production processes, (iii) establishment of advanced analytical and standardization protocols, and (iv) use of computational and systems-based strategies in multi-target drug discovery. More attention needs to be focused on translational validation; whereby promising natural products are tested in clinically relevant systems but not only using in vitro systems.

To sum up, natural products are still invaluable in drug discovery, yet, their full therapeutic potential can be achieved only in a multidisciplinary and translationally oriented way. Through a combination of medicinal chemistry, pharmacology and new technologies, future research will be able to bridge the gap between natural bioactivity and clinical efficacy, and the next generation of therapeutics can be developed using natural sources.

## Figures and Tables

**Table 1:** Classification of principal classes of natural products using representative compounds and structural elements.

Major Class of Natural Products	Biosynthetic Origin	Representative Compounds	Major Therapeutic Applications
Alkaloids	Amino acid-derived pathways	morphine, quinine, berberine	analgesic, antimalarial, antimicrobial, anticancer
Isoprenoids (Terpenoids)	Mevalonate (MVA) and methylerythritol phosphate (MEP) pathways	artemisinin, menthol, paclitaxel	antimalarial, anti-inflammatory, anticancer
Phenolics and Polyphenols	Shikimate and phenylpropanoid pathways	quercetin, resveratrol, curcumin	antioxidant, cardioprotective, anticancer
Flavonoids (Subclass of Polyphenols)	Combined shikimate and malonate pathways	catechin, kaempferol, luteolin	antioxidant, anti-inflammatory, neuroprotective
Steroids	Mevalonate pathway via triterpenoid intermediates	diosgenin, corticosteroids	anti-inflammatory, hormonal regulation
Glycosidic Derivatives of Natural Products	Formed by glycosylation of terpenoids, phenolics, steroids, and other aglycones	digoxin, sennosides, salicin	cardiotonic, laxative, anti-inflammatory
Polyketides	Acetate/malonate pathway	erythromycin, tetracycline	antibacterial, antifungal
Peptides and Non-ribosomal Peptides	Ribosomal and non-ribosomal peptide synthesis	cyclosporine, vancomycin	immunosuppressive, antimicrobial

**Table 2:** Natural products in important medical domains.

Therapeutic Area	Natural Product	Mechanism/Target	Clinical Status	Key Advantages	Major Limitations
Anticancer	Paclitaxel	Microtubule stabilization	Approved	High efficacy	Poor solubility, resistance
Anticancer	Doxorubicin	DNA intercalation, Topo II inhibition	Approved	Broad activity	Cardiotoxicity
Antimalarial	Artemisinin	ROS via endoperoxide cleavage	Approved	Rapid action	Short half-life, resistance
Antibacterial	Vancomycin	Cell wall synthesis inhibition	Approved	Effective vs. Gram+	Nephrotoxicity, resistance
Anti-inflammatory	Curcumin	NF-κB inhibition	Clinical trials	Multi-target	Low bioavailability
Cardiovascular	Digoxin	Na^+^/K^+^/ATPase inhibition	Approved	Strong efficacy	Narrow therapeutic index
Lipid-lowering	Lovastatin	HMG-CoA reductase inhibition	Approved	Proven benefit	Muscle toxicity risk
Neuroprotective	Galantamine	AChE inhibition	Approved	CNS activity	Moderate efficacy
Antidiabetic	Berberine	AMPK activation	Clinical/preclinical	Metabolic regulation	Variable bioavailability

**Table 3:** Key pharmacophoric features across classes.

Class	Key Pharmacophore Features	Representative Insight
Alkaloids	Protonatable nitrogen + aromatic ring	Enables receptor binding
Flavonoids	Phenolic OH + planar ring system	Antioxidant and enzyme interaction
Terpenoids	Hydrophobic core + oxygenated groups	Membrane interaction and target binding
Glycosides	Sugar moiety + active aglycone	Modulates solubility and PK (Pharmacokinetics)
Polyketides	Macrocycle + carbonyl groups	Enzyme inhibition and binding

**Table 4:** Chemical modifications and semisynthetic derivatives.

Parent Compound	Derivative	Modification Strategy	Pharmacokinetic Impact	Therapeutic Impact
Paclitaxel	Docetaxel	Side-chain modification	↑ Solubility, stability	↑ Anticancer efficacy
Artemisinin	Artesunate	Esterification	↑ Bioavailability	↑ Antimalarial activity
Morphine	Codeine	Methylation	↑ Oral absorption	↓ Potency, altered safety
Camptothecin	Irinotecan	Prodrug formation	↑ Stability	↑ Clinical usability
Lovastatin	Simvastatin	Side-chain modification	↑ Potency	Improved lipid lowering

**Table 5:** Pharmacokinetic and toxicity profiles of selected natural products.

Compound	Oral Bioavailability	Metabolism	Half-Life (Approx.)	Toxicity Concern
Curcumin	Low	Rapid glucuronidation	Short	Minimal
Resveratrol	Low	Extensive metabolism	Short	Minimal
Doxorubicin	Moderate	Hepatic metabolism	~20–48 h	Cardiotoxicity

## Data Availability

Where no new data was created.

## Abbreviations

ADMET	Absorption, Distribution, Metabolism, Excretion, and Toxicity
BGC	Biosynthetic Gene Cluster
CD	Circular Dichroism
CNS	Central Nervous System
GC-MS	Gas Chromatography-Mass Spectroscopy
HPLC	High- Performance Liquid Chromatography
HRMS	High-Resolution Mass Spectrometry
ICH	International Council for Harmonization
LC-MS/MS	Liquid Chromatography-tandem Mass Spectrometry
NMR	Nuclear Magnetic Resonance
ORD	Optical Rotatory Dispersion
PK	Pharmacokinetics
PD	Pharmacodynamics
PCA	Principal Component Analysis
PLS-DA	Partial Least Squares Discriminant Analysis
QSAR	Quantitative Structure–Activity Relationship
RP-HPLC	Reversed-Phase High Performance Liquid Chromatography
SAR	Structure–Activity Relationship
VCD	Vibrational Circular Dichroism
